# Clinical Outcomes and Recurrence Rates After Arthroscopic Soft Tissue Stabilization With Selective Augmentations for Traumatic Anterior Shoulder Instability in Athletes Versus Non-athletes With Subcritical Glenoid Bone Loss

**DOI:** 10.7759/cureus.76930

**Published:** 2025-01-05

**Authors:** Kyosun Hwang, Jin Hyeok Lee, Kanghun Yu, Woong Kyo Jeong

**Affiliations:** 1 Orthopaedic Surgery, Korea University Anam Hospital, Seoul, KOR

**Keywords:** arthroscopy, athletes, bankart lesions, joint instability, shoulder

## Abstract

Introduction

Although some studies have reported good outcomes of soft tissue procedures in athletes, to our knowledge, no study has directly compared the clinical outcomes and recurrence rates of soft tissue procedures between athletes and non-athletes. Therefore, we aimed to compare clinical outcomes and recurrence rates between athletes and non-athletes with subcritical glenoid bone defects, who received arthroscopic soft tissue stabilization surgery for traumatic anterior shoulder instability.

Methods

This retrospective comparative study included patients who received primary arthroscopic shoulder stabilization surgery for traumatic anterior shoulder instability, with a minimum two-year follow-up and a glenoid bone defect <20%. The patients were categorized into athlete or non-athlete groups. For the included patients, we performed one of the following procedures: arthroscopic Bankart repair (ABR), ABR with Hill-Sachs remplissage (HSR), or arthroscopic bony Bankart repair (ABBR). We investigated patient characteristics, including preoperative glenoid and humeral head pathology, and compared the two groups in terms of the Korean Shoulder Score for Instability (KSSI), University of California Los Angeles (UCLA) score, Rowe score, and range of motion (ROM) at two years postoperatively. Postoperative recurrence and reoperation rates were also compared.

Results

Altogether, 39 non-athletes and 15 athletes were included. Significantly higher KSSI (athletes: 98.00; non-athletes: 94.64; p = 0.012) was observed in the athletes than in the non-athletes. Meanwhile, the UCLA (athletes: 34.33; non-athletes: 33.87; p = 0.370) and Rowe (athletes: 96.67; non-athletes: 96.67; p = 0.460) scores were not significantly different between the two groups. Five (13%) non-athletes and one (7%) athlete experienced postoperative instability recurrence, which was not significantly different (p = 1.000). Fourteen (93%) athletes returned to sports completely after 5.4 months (range, 4-8 months) on average.

Conclusion

Arthroscopic soft tissue stabilization surgery yielded good clinical outcomes and low recurrence rates in both athletes and non-athletes with subcritical glenoid bone defects.

## Introduction

Traumatic anterior shoulder instability is a prevalent injury among athletes, particularly those involved in high-contact sports such as rugby and American football. This condition not only leads to prolonged absence from sports, but also significantly diminishes athletic performance [1]. Various surgical interventions are available to treat shoulder instability, with the most common being arthroscopic Bankart repair (ABR) and arthroscopic bony Bankart repair (ABBR). These procedures focus on soft-tissue stabilization and are widely accepted for treating traumatic anterior shoulder instability. Despite their popularity, numerous studies have reported high recurrence rates of approximately 6.5%-58% following these surgeries, especially in collision athletes. Thus, more effective and reliable treatment options are warranted [2].

Bone block procedures, such as the Bristow or Latarjet procedures, have gained popularity over soft tissue procedures because of their lower recurrence rates [3,4]. Despite the favorable outcomes of these procedures reported in previous studies, potential complications and reoperation risks are major concerns, suggesting the necessity of stratifying candidates for bone block procedures [4,5]. Moreover, arthroscopic augmentation procedures, such as Hill-Sachs remplissage (HSR) and rotator interval closure, have been suggested as alternatives to bone block procedures [6,7]. Although bone block procedures should be recommended for patients with critical glenoid bone defects without bony fragments [8,9], some studies have reported good outcomes of soft tissue procedures combined with augmentation procedures, such as HSR, in collision athletes with subcritical glenoid bone defects [10,11]. In line with these studies, we have performed soft tissue stabilization procedures using augmentation methods, including HSR and ABBR, in both athletes and non-athletes.

To the best of our knowledge, no study has directly compared the outcomes of soft tissue stabilization procedures between athletes and non-athletes. This study primarily explored whether clinical outcomes or recurrence rates would differ between patients with different activity levels, who were treated using the same surgical decision strategy. Therefore, this retrospective study aimed to compare the clinical outcomes and recurrence rates between athletes and non-athletes who underwent arthroscopic soft tissue procedures for traumatic anterior shoulder instability with subcritical glenoid bone defects. We hypothesized that soft tissue stabilization procedures would yield similarly good clinical outcomes and low recurrence rates in both athletes and non-athletes.

This article was previously presented as a meeting abstract at the 2024 Orthopaedic Summit, on September 14, 2024.

## Materials and methods

This retrospective comparative study was conducted at a single institution and approved by the Institutional Review Board of the Korea University Medical Center (IRB approval no. 2024AN0356).

Patient selection

**Figure 1:**
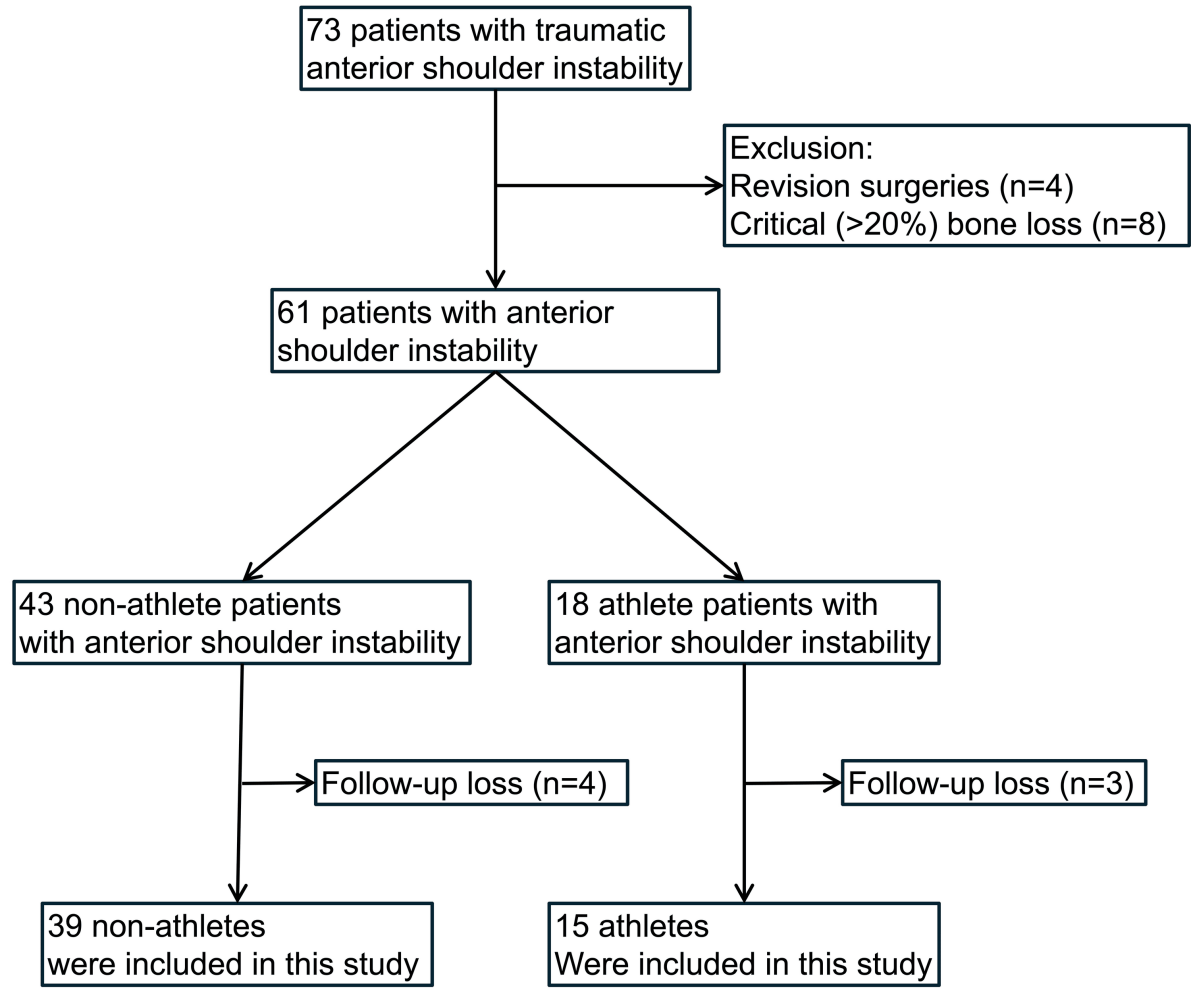
Flowchart of patient selection

Data from patients who underwent arthroscopic stabilization for traumatic anterior shoulder instability at our institute between January 2019 and April 2022 were retrospectively analyzed. All surgical procedures were performed by a single surgeon at our institute. The inclusion criteria were traumatic anterior shoulder instability, arthroscopic stabilization, and primary surgery. The exclusion criteria were as follows: critical (>20%) glenoid bone loss, requiring bone block procedures such as the Latarjet procedure; revision surgeries; and a follow-up period of less than two years (Figure 1).

Data collection

We reviewed the medical records of the included patients, and the patients were divided into professional athlete and non-athlete groups. Preoperative data on patient characteristics, including sex, age at surgery, direction of the affected shoulder, types of sports, number of preoperative dislocations or subluxations, instability severity index score (ISIS) [12], and follow-up periods, were analyzed. Surgical procedures were reviewed and categorized as ABR, ABR combined with HSR, and ABBR. Data on patient-reported outcomes at two years postoperatively were collected, including scores on the visual analog scale (VAS) for pain, Rowe, University of California Los Angeles (UCLA), and Korean Shoulder Score for Instability (KSSI). The range of motion (ROM) of forward elevation (FE) and external rotation at the side (ERSD) was measured in degrees. The ROM of internal rotation (IR) was defined as the highest vertebral level that could be achieved. This was converted into a 10-point scale for analysis, as described in a previous study [13], as follows: buttock-greater trochanter, 2; sacrum-L4, 4; L3-L1, 6; T12-T8, 8; and T7-T1, 10. We also investigated the number of patients who experienced postoperative recurrence of shoulder instability, including dislocation and subluxation, and those who received revision surgery.

Radiographic measurements

We assessed the preoperative glenoid and humeral head pathologies by reviewing preoperative three-dimensional (3D) reconstructed computed tomography (CT) images. Additionally, we calculated the percentage of glenoid bone loss using a previously described circle method, and glenoid morphology was classified into normal, attritional, or bony Bankart [14-16]. Hill-Sachs lesions were evaluated by measuring the maximum depth and width of the lesions on axial CT images. The lesions were also classified as on- or off-track lesions, based on the glenoid track concept described by Yamamoto et al. to assess the engagement of the Hill-Sachs lesions [17].

Surgical procedures

**Figure 2:**
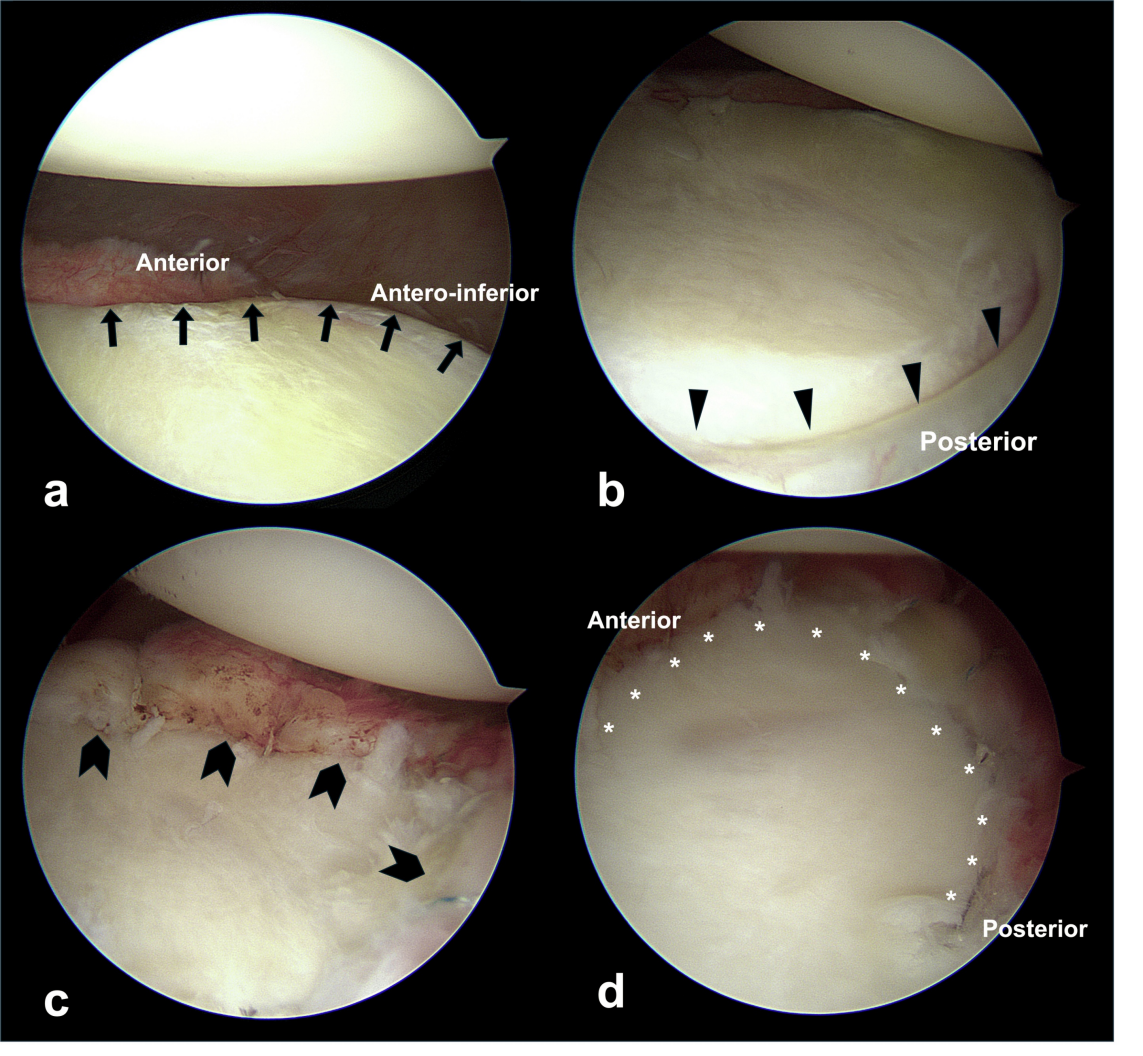
Arthroscopies showing a labral lesion before (a-b) and after arthroscopic Bankart repair (ABR) (c-d) Arrows in (a) indicate loss of labral height from the anterior to anteroinferior portions of the glenoid, and arrowheads in (b) show a detached and depressed posterior labrum. Whole labral lesions were repaired during arthroscopic Bankart repair (ABR) (chevrons) (c), resulting in a circumferentially restored labroligamentous buttress (asterisk) (d).

**Figure 3:**
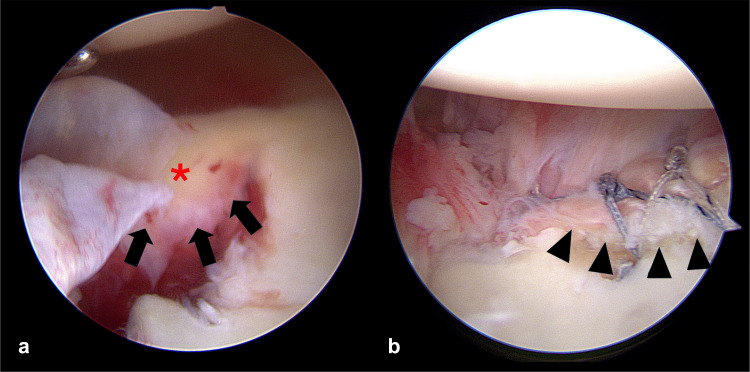
Arthroscopies showing a large bony Bankart lesion (*) before (a) and after (b) arthroscopic bony Bankart repair (ABBR) Arrows show the margin of a detached glenoid fragment which was repaired using the double-row technique (arrowheads).

**Figure 4:**
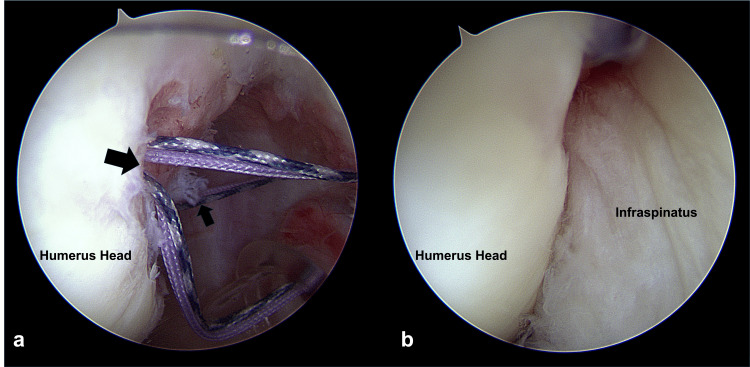
Arthroscopies showing a Hill-Sachs lesion before (a) and after (b) a Hill-Sachs remplissage (HSR) procedure Two suture anchors (arrows) were used for Hill-Sachs remplissage (HSR) by the double-pulley technique.

All surgical procedures were performed with the patient in the semi-lateral position. Routine diagnostic arthroscopy of the glenohumeral joint was conducted, and the labral lesion location was identified. During ABR, the intraoperatively identified labral lesions were completely released from the glenoid margin, which was refreshed before repair. Posteroinferior labral lesions were also repaired, if present, to restore the circumferential labroligamentous buttress (Figure 2). Anteroinferior to posteroinferior labral lesions were repaired using 1.4-mm suture anchors. Although the location of the suture anchors was determined according to the extent of the labral lesion in each shoulder, we always inserted a suture anchor at the 6 o'clock position in all the shoulders analyzed in our study, regardless of the extent of the labral lesion, to maximize glenoid concavity. The anterosuperior labral lesions above the equator of the glenoid were repaired. During ABBR, bony Bankart lesions were repaired using different methods, depending on their size. Small bony Bankart lesions were repaired by suturing the adjacent labroligamentous complex together using simple stitches, whereas medium and large lesions were repaired using the modified Mason-Allen method and double-row repair technique, respectively (Figure 3) [18]. HSR was performed through double-pulley repair, as previously described [9]. Shoulders with off-track Hill-Sachs lesions identified preoperatively were considered for HSR and assessed for engagement under dynamic intraoperative examination with the arm in abduction and external rotation [9]. HSR was performed if engagement was present, even after the repair of all labral or bony Bankart lesions (Figure 4).

Rehabilitation protocol

Passive and assisted active exercises were initiated four weeks after immobilization with an abduction brace. Patients proceeded to the strengthening program 12 weeks postoperatively. During this period, the athletes started sports practice and were permitted to practice more rigorously at 16 weeks postoperatively. Full return to sports was allowed five months postoperatively, depending on the recovery status of each patient.

Statistical analysis

The Shapiro-Wilk test was conducted to test the normality of the data. Student’s t-test or the Mann-Whitney U test was used to compare patient characteristics and clinical outcomes according to the normality of the data. Chi-square and Fisher’s exact tests were performed to compare categorical data, such as postoperative recurrence and reoperation rates. Significance was set at a two-sided p-value of <0.05. All analyses were performed using IBM SPSS Statistics for Windows, Version 27 (Released 2020; IBM Corp., Armonk, NY, USA).

## Results

Patient characteristics

In total, 54 patients who received primary arthroscopic stabilization surgery for traumatic anterior shoulder instability were included in this study (Figure 1). Among these patients, 15 were athletes (12 men and 3 women) with a mean age of 21.3 years (range, 16-32) at surgery. The remaining 39 non-athletes comprised 33 men and 6 women, with a mean age of 27.2 years (range, 18-50). The athletes were significantly younger (p = 0.026) and had significantly higher ISIS (athletes: 5.1; non-athletes: 3.0; p = 0.002) than the non-athletes. Sex, direction of the affected shoulder, number of dislocations or subluxations prior to surgery, and mean follow-up period were not significantly different between the two groups. Among the athletes, seven were ice hockey players, comprising the majority (47%) of the athlete group, and the remaining athletes participated in soccer, judo, and other sports (Table 1).

**Table 1:** Patient characteristics *Statistically significant

			No. (%) or mean (range)		
			Athletes (n = 15)	Non-athletes (n = 39)	p-value
Sex					0.696
Male			12 (80)	33 (85)	-
Female			3 (20)	6 (15)	
Age at surgery, years			21.3 (16-32)	27.2 (18-50)	0.026*
Affected shoulder					0.684
Dominant			9 (60)	21 (54)	-
Nondominant			6 (40)	18 (36)	
Type of sports					-
Ice hockey			7 (47)	-	
Soccer			2 (13)		
Judo			2 (13)		
American football			1 (7)		
Basketball			1 (7)		
Badminton			1 (7)		
Swimming			1 (7)		
No. of dislocations/subluxations prior to surgery			4.9 (0-20)	3.9 (0-50)	0.17
Instability severity index score			5.1 (2-8)	3.0 (0-7)	0.002*
Follow-up period, months			29.9 (24-36)	27.2 (24-48)	0.226

Preoperative pathology

Preoperative 3D CT scans showed significant differences in glenoid bone loss between athletes (11.45%, range 5.8%-18.3%) and non-athletes (6.39%, range 0%-18.9%) (p = 0.008). Glenoid morphology also varied significantly, with 80% of athletes showing attritional changes compared with 46% of non-athletes (p = 0.034), and no athletes had normal glenoid morphology, compared with 36% of non-athletes (p = 0.034). Bony Bankart lesions were present in 20% and 18% of athletes and non-athletes, respectively (p = 1.000). The Hill-Sachs interval (athletes: 14.91 mm; non-athletes: 13.03 mm; p = 0.139) and depth (athletes: 3.03 mm, non-athletes: 3.89 mm; p = 0.059) did not significantly differ between the two groups. Most Hill-Sachs lesions were on track in both groups (athletes: 12, or 80%; non-athletes: 37, or 95%; p = 0.124) (Table 2).

**Table 2:** Preoperative glenoid and humeral head pathologies *Statistically significant

		No. (%) or mean (range)		
		Athletes (n = 15)	Non-athletes (n = 39)	p-value
Glenoid bone loss, %		11.45 (5.8-18.3)	6.39 (0-18.9)	0.008*
Glenoid morphology				-
Normal		0 (0)	14 (36)	0.034*
Attritional		12 (80)	18 (46)	0.034*
Bony		3 (20)	7 (18)	1
Hill-Sachs interval, mm		14.91 (9.7-21.0)	13.03 (0-26.5)	0.139
Hill-Sachs depth, mm		3.03 (1.4-5.1)	3.89 (0-7.4)	0.059
Location of Hill-Sachs lesion				0.124
On-track		12 (80)	37 (95)	-
Off-track		3 (20)	2 (5)	

Types of surgical procedures

Among the 15 athletes, 10 (67%) underwent ABR, three (20%) underwent ABR with HSR, and two (13%) underwent ABBR. In the non-athlete group, 34 (87%) patients underwent ABR, whereas two (5%) and three (8%) patients underwent ABR with HSR and ABBR, respectively. Although more patients in the athlete group underwent additional augmentation procedures than in the non-athlete group, the difference was not significant (Table 3).

**Table 3:** Surgical procedures ABR, arthroscopic Bankart repair; HSR, Hill-Sachs remplissage; ABBR, arthroscopic bony Bankart repair

		No. (%)		
		Athletes (n = 15)	Non-athletes (n = 39)	p-value
Surgical procedures				
ABR		10 (67)	34 (87)	0.119
ABR with HSR		3 (20)	2 (5)	0.124
ABBR		2 (13)	3 (8)	0.61

Clinical outcomes

The mean VAS score at two years postoperatively was 0.53 (range, 0-5) in the athletes and 0.33 (range, 0-1) in the non-athletes, indicating no significant difference (p = 0.528). The KSSI, UCLA, and Rowe scores demonstrated favorable outcomes in both groups. The KSSI score was significantly higher in the athletes (athletes: 98.00; non-athletes: 94.64; p = 0.012) than in the non-athletes. Meanwhile, the UCLA (athletes: 34.33; non-athletes: 33.87; p = 0.370) and Rowe (athletes: 96.67; non-athletes: 96.67; p = 0.460) scores did not differ significantly between the two groups. Compared with the non-athletes, the athletes had a significantly larger mean ROM of the FE (athletes: 178.00; non-athletes: 172.82; p = 0.023) and significantly lower IR (athletes: 7.73; non-athletes: 8.36; p = 0.033). The ROM of the ERSD was not significantly different between the two groups (athletes: 72.33; non-athletes: 68.85; p = 0.504). Fourteen (93%) patients in the athlete group completely returned to their preinjury sports level after an average of 5.4 months (range, 4-8) of postoperative rehabilitation (Table 4).

**Table 4:** Clinical outcomes and postoperative recurrence rates *Measured two years postoperatively; †Statistically significant; ‡Includes patients experiencing postoperative subluxation without true dislocation VAS, visual analog scale; KSSI, Korean shoulder score for instability; UCLA, University of California Los Angeles; ROM, range of motion; FE, forward elevation; ERSD, external rotation at the side; IR, internal rotation; ABR, arthroscopic Bankart repair; HSR, Hill-Sachs remplissage

		No. (%) or mean (range)		
		Athletes (n = 15)	Non-athletes (n = 39)	p-value
VAS, points*		0.53 (0-5)	0.33 (0-1)	0.528
Patient-reported outcomes, points*				
KSSI		98.00 (90-100)	94.64 (66-100)	0.012^†^
UCLA		34.33 (31-35)	33.87 (26-35)	0.37
Rowe		96.67 (90-100)	96.67 (75-100)	0.46
ROM*				
FE, degrees		178.00 (170-180)	172.82 (150-180)	0.023^†^
ERSD, degrees		72.33 (70-80)	68.85 (20-90)	0.504
IR, points		7.73 (6-8)	8.36 (6-10)	0.033^†^
Return to preinjury sports activity, no		14 (93)	-	
Time to sports return, months		5.4 (4-8)		
No. of postoperative recurrence		1 (7)	5 (13)	1
Postoperative dislocation		1 (7)	3 (8)	1
Postoperative subluxation^‡^		0	2 (5)	1
No. of revision surgeries		1 (7)	2 (5)	0.632
Revisional ABR		0	1	-
Revisional ABR with HSR		0	1	
Latarjet procedure		1	0	

Postoperative recurrence rates

No significant differences in the postoperative recurrence rates, including dislocation and subluxation, were observed between the two groups. Postoperative dislocation occurred in only one athlete (7%) and three non-athletes (8%) (p = 1.000). Postoperative subluxation occurred in two non-athletes (5%) and in none of the athletes (p = 1.000). One athlete (7%) and two non-athletes (5%) required revision surgery (p = 0.632). Specifically, revisional ABR alone was performed in one non-athlete, and revisional ABR with HSR was performed in another non-athlete. The Latarjet procedure was performed on only one athlete; none of the non-athletes underwent this procedure (Table 4).

## Discussion

The most important finding of our study was that arthroscopic soft tissue stabilization, based on the concept of a selective augmentation strategy, showed similarly favorable clinical results in athletes with traumatic anterior shoulder instability as in non-athletes. Both athletes and non-athletes also demonstrated consistently low recurrence and revision surgery rates. All athletes, except one who received revision surgery, returned to sports completely after surgery.

One of the most significant concerns in the management of traumatic anterior shoulder instability is deciding whether to perform bone block procedures, such as the Bristow or Latarjet procedure. Glenoid bone loss is one of the most important risk factors for recurrence after soft tissue stabilization procedures, necessitating bone block [19]. Although bone block procedures are widely accepted for critical (>20%) glenoid bone loss [8,9], some studies have reported varying results of soft tissue procedures in athletes with subcritical-range glenoid bone defects [10,20-22]. In our study, all the patients with glenoid bone loss of <20% were treated with soft tissue stabilization procedures, with selective augmentation procedures, regardless of their activity level, which resulted in favorable clinical outcomes with similarly low recurrence and revision rates in both athletes and non-athletes. This result aligns with those of previous studies reporting satisfactory outcomes after arthroscopic soft tissue procedures for traumatic shoulder instability in athletes [10,11]. Considering these favorable results, soft tissue stabilization procedures may ensure stability and prevent recurrence for shoulders with subcritical-range glenoid bone defects, regardless of the activity level, if combined with appropriate selective augmentation procedures targeting individual preoperative pathologic conditions.

ISIS was significantly higher in athletes, which seems plausible because of their higher levels of activity and trauma energy compared with non-athletes [23]. ISIS is a widely accepted method for evaluating the risk of failure after ABR, and bone block procedures are recommended for patients with an ISIS of >6 [12]. Although the significantly higher ISIS observed in the athletes than in the non-athletes in our study raises concerns regarding the higher risk of recurrence after soft tissue procedures for the athletes [24], we did not consider ISIS for the Latarjet procedure. Latarjet procedures were reserved for shoulders with critical (>20%) glenoid bone loss and were excluded from our study. One possible explanation for the favorable results in the athletes despite higher ISIS is that ISIS has some limitations in determining the surgical strategy. The presence of Hill-Sachs lesions and loss of glenoid contour, which are key components of ISIS, were evaluated using plain radiographs. Although plain radiography is a simple and accessible modality, it may not accurately assess glenoid and humeral head morphologies. The glenoid track instability management score (GTIMS), a scoring system recently proposed by Di Giacomo et al. [25], uses CT scans to evaluate on- or off-track Hill-Sachs lesions. The study reported that the ISIS recommended two-fold more patients for the Latarjet procedure compared with the GTIMS, and patients who were arthroscopically treated according to the ISIS showed significantly worse clinical outcomes than those treated arthroscopically according to the GTIMS. Thus, surgical decisions for traumatic anterior instability should not be guided solely by ISIS, and individual pathologies should also be considered.

To the best of our knowledge, no study has directly compared glenoid and humeral head pathologies between athletes and non-athletes. In terms of preoperative glenoid and humeral head pathologies, the percentage of glenoid defects was significantly higher in the athletes than in the non-athletes. Moreover, the mean Hill-Sachs interval and proportion of off-track lesions were higher in the athletes than in the non-athletes, although this was not statistically significant. In contrast to previous studies that have reported higher rates of bony Bankart lesions [11,15], only three (20%) patients in the athlete group and seven (18%) in the non-athlete group had bony Bankart lesions in our study. Despite the similarly low prevalence of bony Bankart lesions in both the athletes and non-athletes in our study, glenoid morphology differed significantly. In particular, 36% of the non-athletes showed normal glenoid morphology preoperatively, compared with the athletes, who had either an attritional glenoid defect or a bony Bankart lesion. A large glenoid defect size and absence of normal glenoid morphology in the athletes in our study suggest that patients with different activity levels have preoperative glenoid pathologies of significantly different severity. Hill-Sachs lesions and glenoid tracks showed no significant differences between the two groups; however, these findings need to be further validated by multi-center studies with larger populations.

Unlike a previous study that has reported performing HSR in more than half of the patients [11], the most frequently performed surgical procedure in our study was ABR, which was performed in 87% of all the procedures in the non-athletes and 67% in the athletes. This discrepancy may be attributed to the different sports types of the athletes in these studies. All the patients included in the previous study were rugby players, whereas, in our study, eight (53%), five (33%), and two (13%) were collision, contact, and non-contact athletes, respectively. One possible explanation for the good ABR results is that we repaired all existing labral lesions, including posterior and inferior lesions, to restore the circumferential height of the labroligamentous complex. The rationale for circumferential labral repair in our study lies in the primary role of the concavity compression mechanism in glenohumeral joint stability [26]. The importance of restoring the glenoid concavity for shoulder stability has been supported by many previous studies, including both biomechanical [27] and clinical studies [28]. These studies explain the good results of our procedures, which were mainly ABR, in the athletes despite higher ISIS and larger glenoid bone defects than in the non-athletes. In addition, more athletes underwent augmentation procedures than non-athletes, which seems congruent with the results in Table 2, showing larger glenoid bone defects, worse glenoid morphology, and more off-track lesions in the athletes, although this difference was not significant. Hence, surgical plans may have been guided appropriately based on the preoperative pathologies, leading to good results in our study. Since activity levels were controlled under the rehabilitation protocol during the postoperative period, preoperative pathologies may be the most important factors that should be considered for surgical decisions to achieve good results and low recurrence rates.

The FE ROM of the athletes was significantly higher than that of the non-athletes. This may have resulted from a more rigorous rehabilitation protocol and better compliance from the athletes compared to the non-athletes. In contrast to this finding, the IR ROM was significantly lower in the athletes than in the non-athletes in our study. This may be attributed to the suboptimal postoperative rehabilitation for IR ROM in athletes. Since throwing athletes, who are prone to glenohumeral internal rotation deficit (GIRD) [29], were not included in our study, concerns regarding IR deficiency may not have been sufficiently considered during the rehabilitation period. Although a previous study by Ohuchi et al. has reported a 10% prevalence of GIRD in contact sports players [30], further studies are needed to explain this result.

This study had some limitations. The distribution of sports types in the athlete group was heterogeneous. Various sports types, including ice hockey, soccer, basketball, football, badminton, and swimming, were included in the athlete group, so it comprised collision, contact, and non-contact athletes for comparison with non-athletes. However, the majority (86%) of the athletes were involved in collision or contact sports, and all the athletes were elite athletes, raising similar concerns about worse clinical results and recurrence after soft tissue stabilization procedures. In addition, the study had a retrospective design and a relatively small sample size. High-quality prospective comparative studies with larger populations are required to confirm the results of the present study. Nevertheless, to the best of our knowledge, this is the first study to compare the outcomes of soft tissue procedures between athletes and non-athletes with traumatic anterior shoulder instability.

## Conclusions

In conclusion, arthroscopic soft tissue stabilization surgery resulted in favorable clinical outcomes and low recurrence rates in both athletes and non-athletes with traumatic anterior shoulder instability. Despite athletes having significantly higher mean ISIS scores, greater glenoid bone loss, and more severe glenoid morphology, they demonstrated better KSSI scores and comparable UCLA and Rowe scores to non-athletes. The majority of athletes successfully returned to sports following surgery. For shoulders with subcritical glenoid bone defects, soft tissue procedures can lead to good clinical outcomes, regardless of activity level, when combined with appropriate augmentation techniques tailored to individual preoperative pathological conditions.
